# A selective eradication of human nonhereditary breast cancer cells by phenanthridine-derived polyADP-ribose polymerase inhibitors

**DOI:** 10.1186/bcr2445

**Published:** 2009-11-09

**Authors:** Dana Inbar-Rozensal, Asher Castiel, Leonid Visochek, David Castel, Françoise Dantzer, Shai Izraeli, Malka Cohen-Armon

**Affiliations:** 1 The Neufeld Cardiac Research Institute and Dept. of Physiology and Pharmacology, Sackler School of Medicine, Tel-Aviv University, Tel-Aviv 69978, Israel; 2 Cancer Research Institute, Sheba Medical Center, Tel-Hashomer, Ramat-Gan 52621, Israel; 3 Laboratory of Molecular and Structural Biology, Ecole Superieure de Biotechnologie de Strasbourg, F-67400, Illkrich-Graffenstaden, France

## Abstract

**Introduction:**

PARP-1 (polyADP-ribose polymerase-1) is known to be activated in response to DNA damage, and activated PARP-1 promotes DNA repair. However, a recently disclosed alternative mechanism of PARP-1 activation by phosphorylated externally regulated kinase (ERK) implicates PARP-1 in a vast number of signal-transduction networks in the cell. Here, PARP-1 activation was examined for its possible effects on cell proliferation in both normal and malignant cells.

**Methods:**

*In vitro *(cell cultures) and *in vivo *(xenotransplants) experiments were performed.

**Results:**

Phenanthridine-derived PARP inhibitors interfered with cell proliferation by causing G_2_/M arrest in both normal (human epithelial cells MCF10A and mouse embryonic fibroblasts) and human breast cancer cells MCF-7 and MDA231. However, whereas the normal cells were only transiently arrested, G_2_/M arrest in the malignant breast cancer cells was permanent and was accompanied by a massive cell death. In accordance, treatment with a phenanthridine-derived PARP inhibitor prevented the development of MCF-7 and MDA231 xenotransplants in female nude mice. Quiescent cells (neurons and cardiomyocytes) are not impaired by these PARP inhibitors.

**Conclusions:**

These results outline a new therapeutic approach for a selective eradication of abundant nonhereditary human breast cancers.

## Introduction

PolyADP-ribose polymerases (PARPs) catalyze a posttranslational and energy-consuming modification of proteins by polyADP-ribosylation. This enzymatic reaction is initiated by ADP-ribose transferase activity, which proceeds with polymerization of ADP-riboses into long and branched polymers [1]. In the chromatin, polyADP-ribosylation apparently regulates the interaction of PARPs and their substrates with protein partners and DNA. PARP-1 is a highly conserved DNA-binding protein and the most abundant nuclear PARP. The enzyme is known to be activated in response to DNA single-strand breaks [1], and its activation induces chromatin remodeling, rendering the DNA more accessible to transcription factors and repair enzymes [1,2].

Our recent findings in quiescent cells and in cell-free systems disclosed an alternative mechanism, inducing an intense and long-lasting activation of PARP-1 in the absence of DNA damage [3,4]. In this process, PARP-1 interaction with phosphorylated ERK2 (externally regulated kinase) resulted in PARP-1 activation and polyADP-ribosylation in a positive-feedback mechanism that kept PARP-1 polyADP-ribosylated as long as ERK2 was phosphorylated [3]. In addition, polyADP-ribosylated PARP-1 highly augmented the activity of phosphorylated ERK, enhancing phosphorylation of ERK-targeted transcription factors, core histone acetylation, and the expression of ERK target genes, some of which are oncogenes [3-6]. Because ERK activity in the nucleus is a key modulator for inducing proliferation versus differentiation in a variety of cancer cells [7,8], these findings suggest that PARP-1 activation might be a possible target for mechanisms inducing cell proliferation.

PARP inhibitors were designed to prevent PARP-1 activation in response to nicked DNA, in an attempt to suppress PARP mediated DNA repair [9-12]. Several generations of PARP inhibitors were designed to prevent PARP-1 activity by blocking the binding of the nicotinamide moiety of NAD^+ ^in the catalytic site of the enzyme. PARP inhibitors differ in their chemical structure, their potency, stability, solubility in water, and apparently even in their therapeutic potential [9-12]. Several groups of PARP inhibitors (including phenanthridine derivatives) were designed to protect cells under stress conditions from cell death induced by a massive activation of PARP-1 (for example, stroke, inflammation; [10,12]), or to cause cell death in malignant cells by preventing polyADP-ribosylation-dependent DNA repair [9,11]. In accordance with this concept, PARP inhibitors were tested for their therapeutic potential in malignant cells with impaired DNA-repair machinery [13,14] (bearing mutations in the tumor-suppressor genes *BRCA1 *and *BRCA2 *that cause an impaired DNA repair [15]) or in combination with DNA-damaging treatments [11]. However, in view of findings indicating that activated PARP-1 highly augments the activity of ERK in the nucleus even in the absence of DNA damage [3,4], a different therapeutic potential of PARP inhibitors is examined in breast cancer cells lacking BRCA mutations.

## Materials and methods

Human breast cancer cell line MCF-7 and MDA231 and human epithelial cells MCF-10A were supplied by ATCC Co. (American Type Culture Collection, P.O. Box 1549, Manassas, VA 20108, USA. The dealer in Israel is Almog Diagnostic & Medical equipment Ltd.). Mouse embryonic fibroblasts were prepared in the laboratory of Dr. Dantzer (Strasbourg, France).

MCF-7 and MDA231 cells were cultured in six-well multidish plates (Nunc, Thermo Fisher scientific, Reskilde, Denmark). MCF-7 and MDA231 cells were maintained in a medium containing DMEM (cat. 01055-1A), 10% Fetal bovine serum (FBS; cat. 04-124-1A), 1% L-glutamine (cat. 03-020-1B), and 1% Pen-Strep Ampho (cat. 03-033-1B) (Gibco, purchased from Rhenium, Rehovot, Israel).

MCF-10A human epithelial cells were cultured in DMEM/F12 (Gibco) with FBS (Gibco) 6%, EGF (100 μg/ml, Cytolab, Rehovot, Israel) 0.02%, hydrocortisone (50 μ*M*, Sigma) 2.8%, insulin (10 mg/ml, Sigma) 0.1%, and Pen/Strep (Gibco) 1%.

Mouse embryonic fibroblasts were cultured in a medium containing DMEM (cat. 01055-1A), 10% FBS (cat. 04-121-1A), 1% L-glutamine (cat. 03-020-1B), and 1% Pen-Strep Ampho (cat. 03-033-1B) (Gibco, purchased from Rhenium, Rehovot, Israel).

### Phenanthridine-derived PARP inhibitors

**Figure 1:**
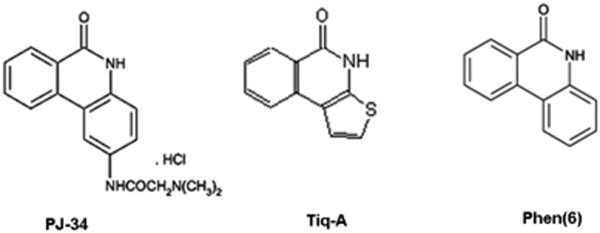
The chemical structures of the PARP inhibitors PJ-34, Tiq-A, and Phen.

We examined the effect of phenanthridine-derived compounds that act as potent PARP inhibitors. These included PJ-34, (*N*-(6-oxo-5,6-dihydrophenanthridin-2-yl)-*N, N*-dimethyl-acetamide), Tiq-A, (4H-thieno [2,3-c]isoquinolin-5-one) and Phen, (6(5H) phenanthridinone). PJ-34 and Phen were purchased from Alexis Biochemicals (Alexis Corporation, Lausanne, Switzerland). Tiq-A was purchased from Sigma, Israel. Their chemical structure is presented in Figure 1.

### The effect of PJ-34 on the development of MCF-7 and MDA231 xenotransplants

Xenotransplants were developed in female CD-1 *nu/nu *mice 5 to 6 weeks old (Charles River Labs, Sulzfeld, Germany) purchased in Israel from Harlan Labs, Jerusalem. MCF-7 and MDA231 cells were injected subcutaneously, about 10^7 ^MCF-7 or MDA231 cells in 150 μl of PBS and 150 μl of Matrigel Basement Membrane Matrix (Becton Dickinson, Bedford, MA, USA; In Israel, Bactolab Diagnostics). In mice treated with PJ-34, Injection was adjacent to subcutaneous osmotic pumps dripping PJ-34 by a slow release. The mice were maintained under specific pathogen-free conditions with access to mouse chow and water *ad libitum*. PJ-34 (2 m*M *dissolved in 100 μl PBS) was inserted in subcutaneously implanted Alzet osmotic pumps, designed to release PJ-34 continuously (at about 0.6 nmol/h) for 14 days. For comparison, in the *in vitro *experiments, the amount of PJ-34 per dish was approximately 20 nmol. Subcutaneous implantation of these pumps was performed before injection by a veterinarian (Dr. Kastel David). All the experiments with nude mice conform with the *Guide for the Care and Use of Laboratory Animals *published by the NIH (publication No. 85-23, revised 1996). Approval was granted by the Israeli Ministry of Health ethics review board in the Tel-Aviv University (M08033).

### Cell survival in cells treated with PJ-34

MCF-7 or MDA231 cells (seeded about 500,000 cells/3-mm well in six-well plates) were treated with PJ-34 applied only once, 24 hours after seeding. In some experiments, cells were reseeded in PJ-34-free medium in 10-cm plates for colony formation. In these experiments, after 2 weeks of incubation without application of PJ-34, cells were fixed (methanol/acetic acid, 3:1), stained with crystal violet, and counted to determine cell survival.

Flow cytometry was used to monitor changes in the ploidy level of malignant and normal cells labeled with propidium Iodide (PI) staining. Counting the cells with flow cytometry in selected time periods for several hours, indicated changes in their cell cycle caused by treatment with PJ-34. Flow cytometry was performed by using a Becton Dickenson machine and the FlowJo software (Tree Star, Ashland, OR, USA). Untreated cells were used as controls for each cell type.

## Results

### Phenanthridine-derived PARP inhibitors efficiently eradicated MCF-7 and MDA231 breast cancer cells without impairing human epithelial MCF-10A or mouse embryonic fibroblasts

**Figure 2:**
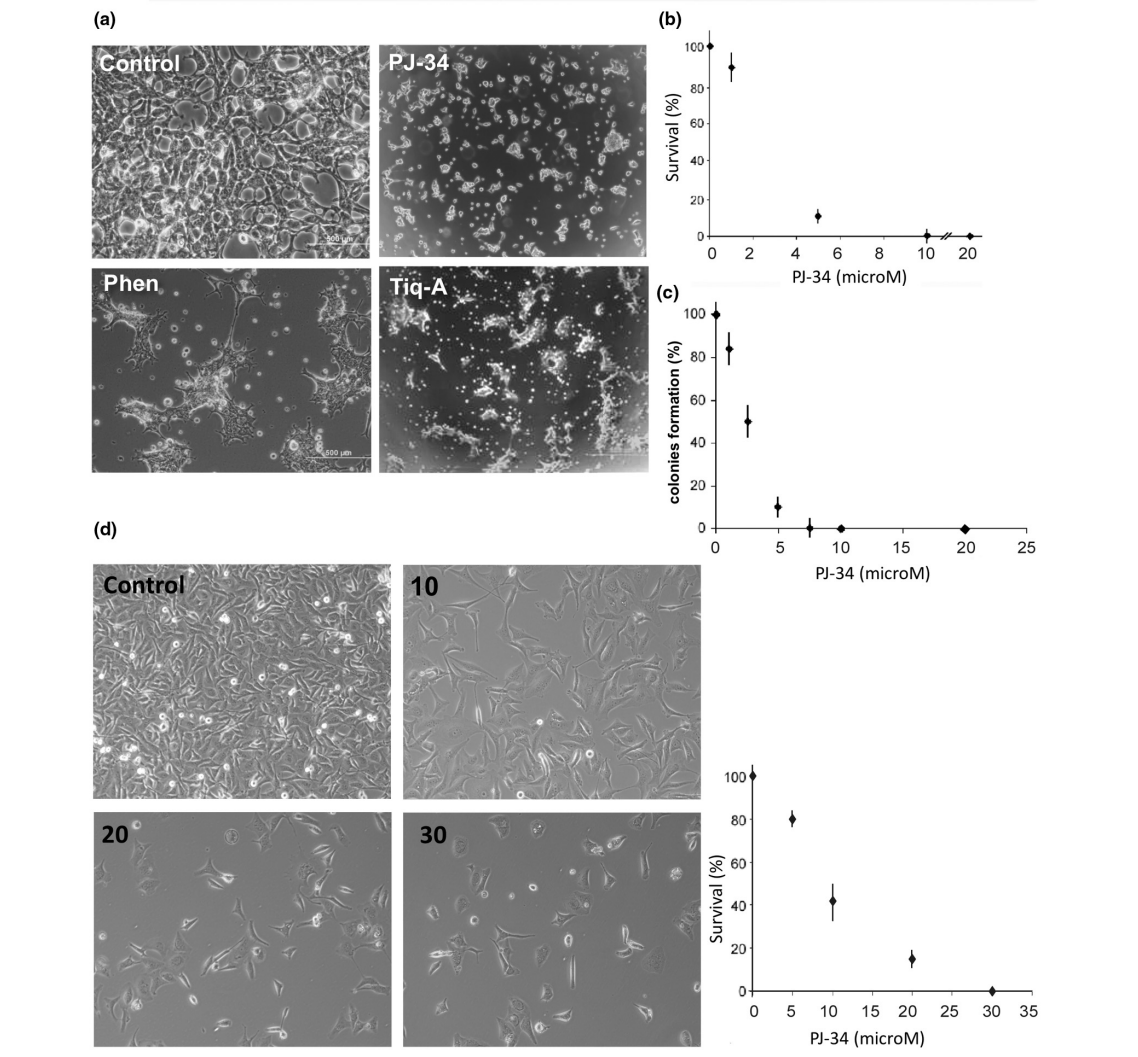
Eradication of MCF-7 and MDA231 human breast cancer cells by phenanthridine-derived PARP inhibitors. **(a) **MCF-7 cells were seeded (about 500,000/well) in six-well plates and incubated for 48 hours with the potent PARP inhibitors, PJ-34 (10 μM), Tiq-A (50 μM), and Phen (25 μM), each applied (a single application) 24 hours after seeding. Cells were counted and pictured under a microscope (0.02 to 0.03 mm^2 ^per field). Control (upper left) and treated MCF-7 cells are presented, 72 hours after seeding. These representative results were repeated in five different experiments. **(b) **Survival rate (percentage relative to untreated cells at each time point, control) of MCF-7 breast cancer cells after incubation for 48 hours with several concentrations of PJ-34, applied (a single application) 24 hours after seeding. Each value represents the average value of five measurements in different cell cultures. **(c) **Colony formation (percentage relative to colony formation of untreated cells) of MCF-7 breast cancer cells reseeded and incubated for 2 additional weeks in the absence of PJ-34, 48 hours after a single application of PJ-34 at several concentrations was applied 24 hours after the initial seeding (Methods). Each value represents the average value of three measurements in three different experiments. **(d) Left panel: **MDA231 breast cancer cells were seeded (about 500,000/well) in six-well plates. PJ-34 was applied into the medium at the indicated final concentrations 24 hours after seeding. Cells were counted and pictured under microscope (0.02 to 0.03 mm^2 ^per field). **Right panel: **Survival rate (percentage relative to untreated cells at each time point, control) of MDA231 breast cancer cells after 72 hours of incubation with several concentrations of PJ-34, applied (a single application) 24 hours after seeding. Each value is an average of three measurements obtained in three different experiments.

We examined the effect of PARP inhibitors on MCF-7 and MDA231 human breast cancer cells. Cells were treated with the potent PARP inhibitors, PJ-34, Tiq-A, and Phen (Figure 1) applied only once, 24 hours after seeding. MCF-7 cells did not survive after 48 to 72 hours of incubation with 10 μM PJ-34 (Figure 2a), nor after incubation with Tiq-A (100 μM) or Phen (50 μM) (Figure 2a). At these concentrations, PJ-34, Tiq-A, and Phen inhibit the activity of PARP-1 [3,10,16,17]. Significant cell death was observed even at lower concentrations of PJ-34 (Figure 2b). More than 99% of MCF-7 cells were eradicated after 48 hours of incubation with 10 μM PJ-34 (Figure 2b). The damage was irreversible. No recovery was observed in MCF-7 cells treated with PJ-34 for 48 to 72 hours and then reseeded in PJ-34-free medium and incubated for 2 additional weeks in the absence of PJ-34 (Figure 2c). Massive cell death also was observed in MDA231 incubated for 72 to 96 hours with PJ-34 applied only once, 24 hours after seeding. These cells were completely eradicated by incubation with 20-30 μM PJ-34 (Figure 2d). No recovery was observed in MDA231 cells incubated with 30 μM PJ-34 for 72 hours, after reseeding in PJ-34-free medium and incubation for 2 additional weeks.

**Figure 3:**
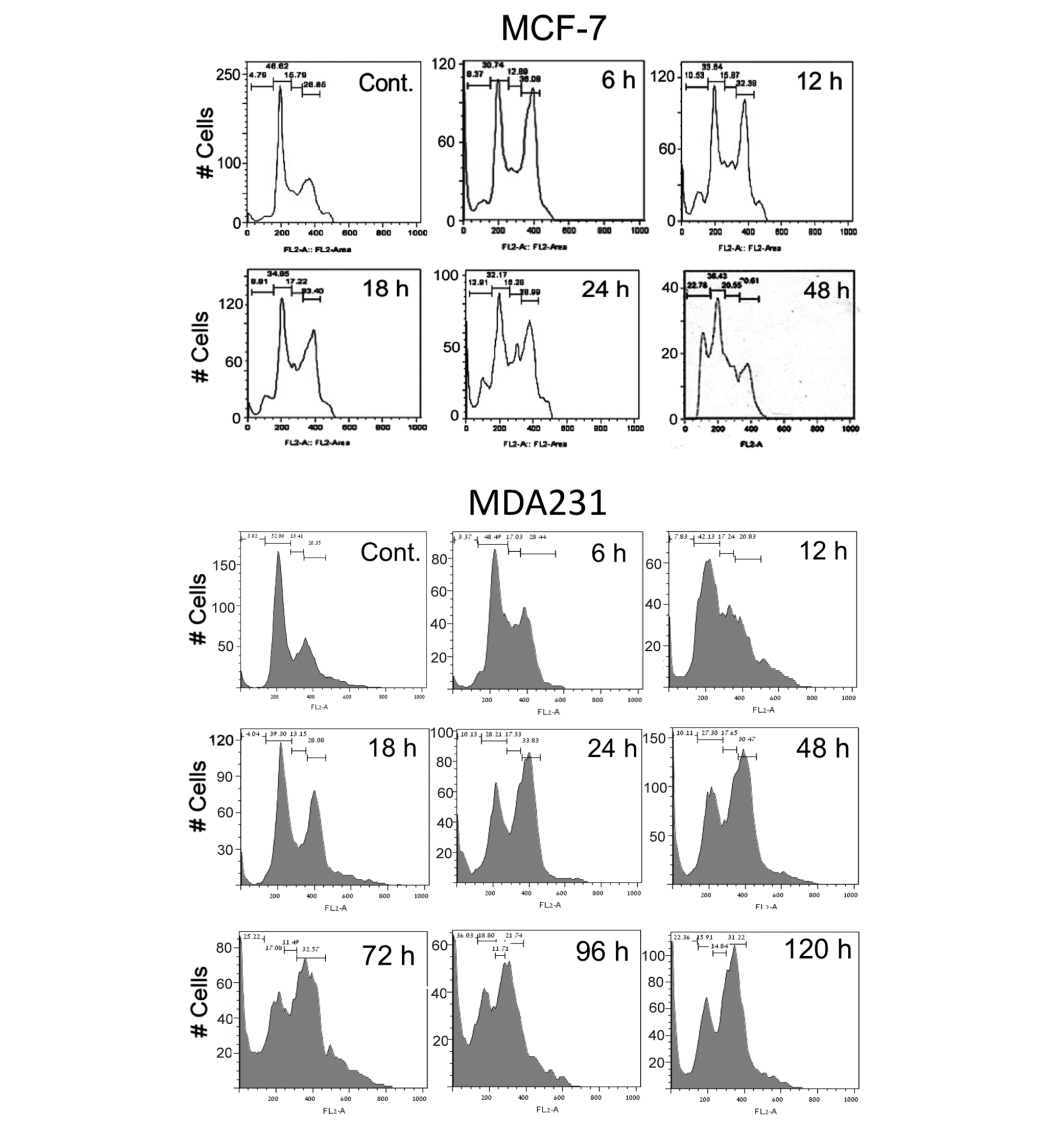
G_2_/M arrest and cell death in MCF-7 and MDA231 treated with PJ-34. The effect of PJ-34 on the cell cycle was examined with flow cytometry. PJ-34 (10 μM) was applied to both types of cells, 24 hours after seeding. Controls: Untreated MCF-7 48 hours after seeding and untreated MDA231 cells 72 hours after seeding. At the indicated time, cells were collected, permeabilized (75% ethanol in DDW), and stained with propidium iodide (PI). The effects of PJ-34 on cell eradication and the kinetics of S-phase entry and G_2_/M transition were evaluated by the percentages of cells at these phases. G_2_/M arrest accompanied by cell death was detected in both cell types after 6 hours of incubation with PJ-34. Similar results for both cell types were obtained in three different experiments.

Flow cytometry disclosed G_2_/M arrest and cell death in both MCF-7 and MDA231 cells. G_2_/M arrest was already observed in both cell types 6 hours after treatment with 10 μM PJ-34. It was not relieved within 120 hours of the experiment and was accompanied by massive cell death (Figure 3).

**Figure 4:**
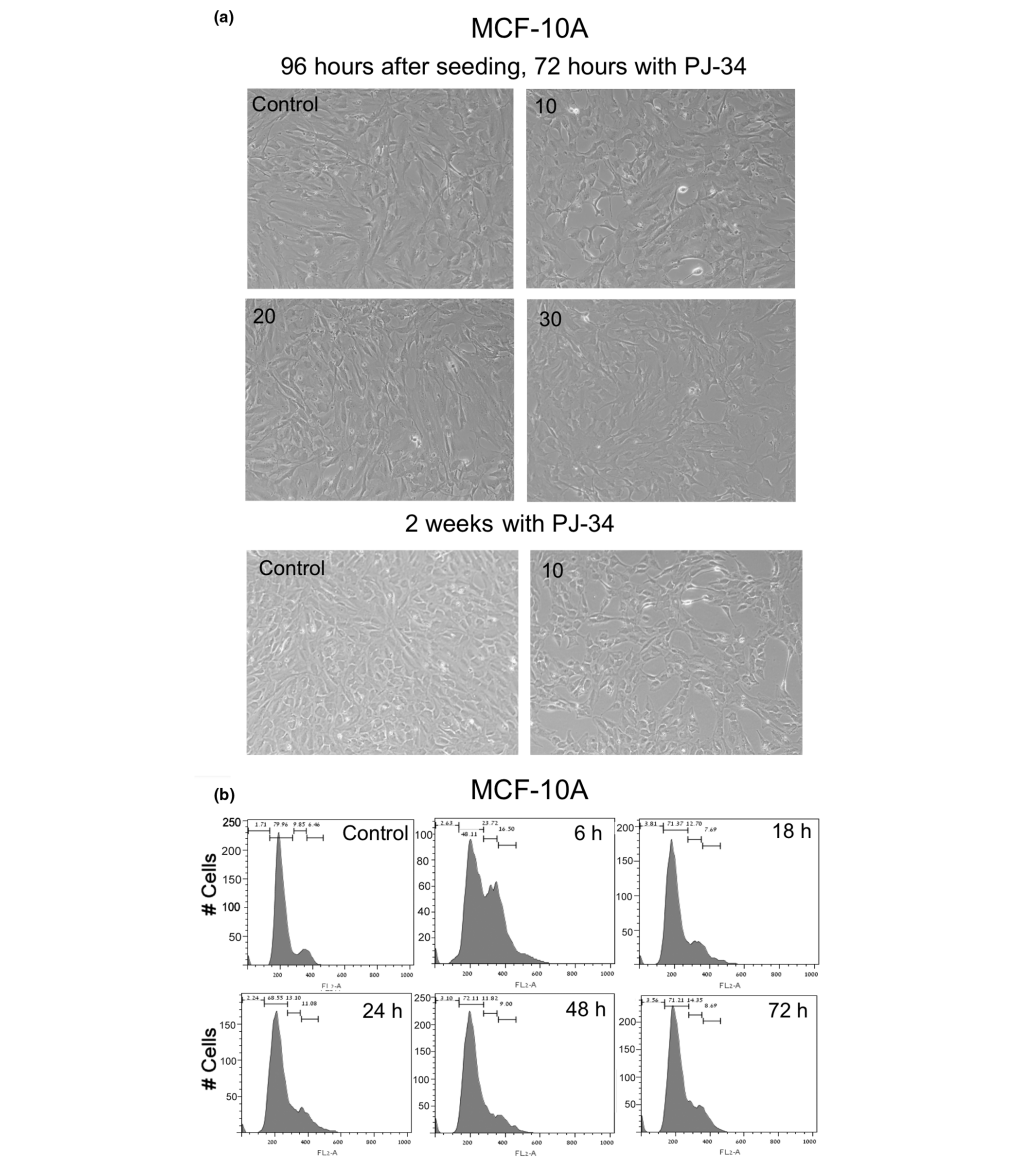
PJ-34 did not eradicate normal human epithelial cells MCF-10A. **(a) **MCF-10A cells were seeded (about 500,000/well) in six-well plates (Methods). PJ-34 was applied once at the indicated concentrations to MCF-10A cells 24 hours after seeding. Untreated MCF-10A cells and MCF-10 cells incubated with 10, 20, and 30 μM PJ-34 for 72 hours, and MCF-10A cells incubated with 10 μM PJ-34 for 2 weeks, were pictured under a microscope. These representative results were observed in three different experiments. **(b) **MCF-10A cells overcame G_2_/M arrest induced by treatment with PJ-34 (10 μM). Cells were analyzed with flow cytometry at the indicated periods after addition of 10 μM PJ-34. G_2_/M arrest detected 6 hours after PJ-34 application was relieved after 18 hours. Control represents untreated MCF-10A cells 72 hours after seeding. Similar results were measured in three different experiments.

Normal dividing cells, human epithelial cells MCF-10A (Figure 4a), were similarly arrested at G_2_/M (Figure 4b). Their arrest also was detected 6 hours after application of PJ-34 (10 μM). However, unlike the malignant cells, MCF10A cells were only temporarily arrested (no arrest observed after 18 hours of incubation with PJ-34), and this transient arrest was not accompanied by cell death (Figure 4a and 4b). MCF-10A cells overcame the cell-cycle arrest, and continued to proliferate as normal cells, even when incubated with the same concentrations of PJ-34 and for the same durations used to eradicate MDA231 cells (compare Figures 2d and 4a). Also, proliferation of MCF-10A cells was not significantly reduced, even after a long incubation of 14 days with 10 μM PJ-34 (Figure 4a).

**Figure 5:**
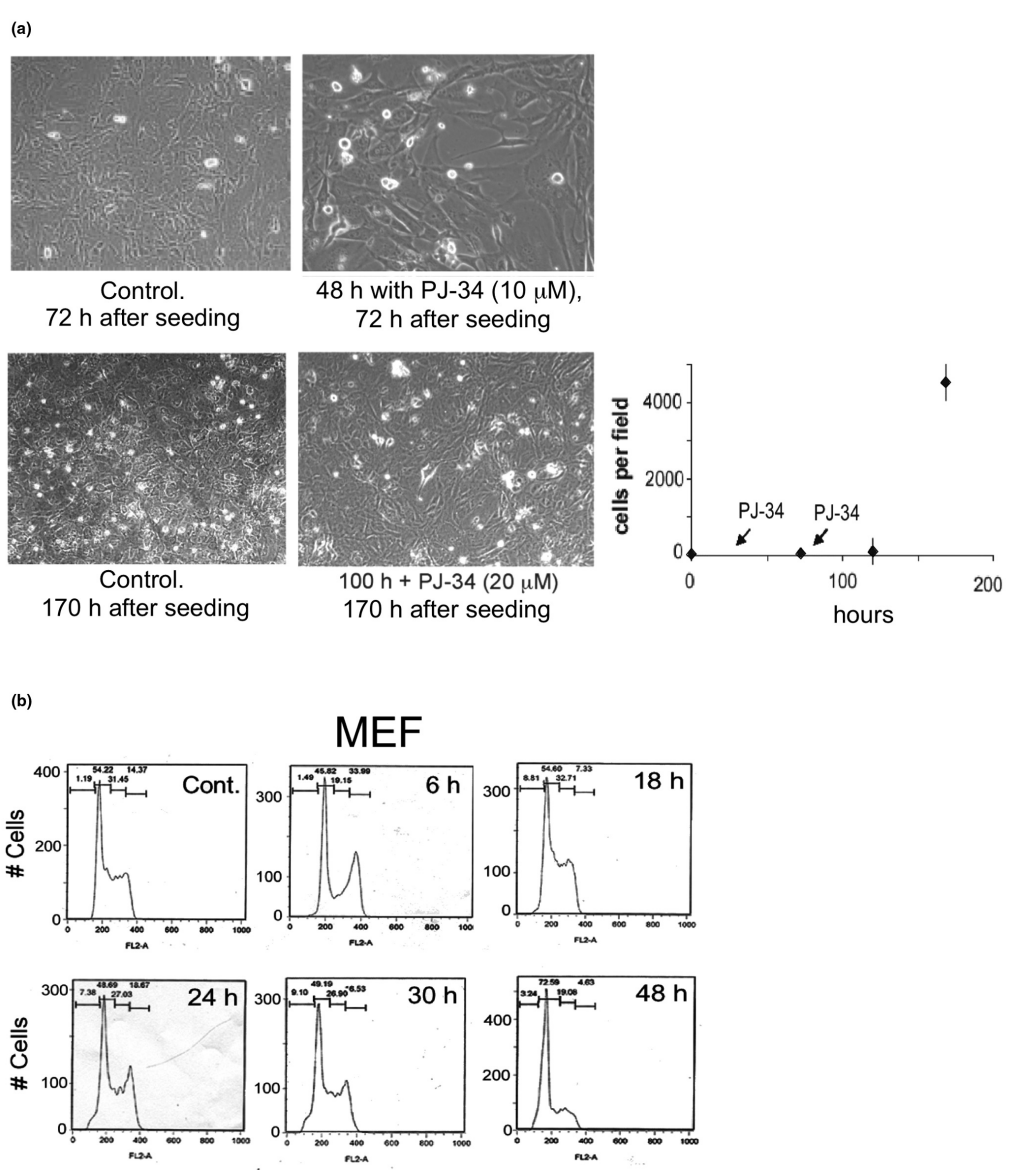
PJ-34 did not eradicate mouse embryonic fibroblasts. **(a) **The effect of PJ-34 on mouse embryonic fibroblasts (MEF) was measured after repeated applications of PJ-34 (10 μM) 24 hours and 72 hours after seeding, as indicated. MEF, untreated and incubated with PJ-34 (10 μM) for 48 hours, and MEF, untreated and incubated with 20 μM PJ-34 for 100 hours are shown. On the right are displayed cell counts (0.02 to 0.03 mm^2 ^per field) of cells incubated with PJ-34 (10 μM, after the first application, and 20 μM, after the second application) for the indicated time periods. Each value is an average value of cells counted in five different experiments. **(b) **MEFs were analyzed with flow cytometry at the indicated periods after addition of PJ-34. A transient G_2_/M transition arrest is indicated in MEFs incubated for 6 hours with PJ-34 (10 μM). Control: Untreated MEFs 48 hours after seeding. Similar results were obtained in three different experiments.

G_2_/M cell-cycle arrest also was detected in mouse embryonic fibroblasts (Figure 5a) after 6 hours of incubation with PJ-34 (10 μM) (Figure 5b). These cells also overcame the cell-cycle arrest, and the arrest in cell cycle was not accompanied by cell death (Figure 5b).

Thus, treatment with PJ-34 induced a transient G_2_/M arrest in these normal proliferating cells, which was not accompanied by cell death (Figures 4 and 5), whereas the cell cycle of malignant cells MCF-7 and MDA231 was permanently arrested, and these cells were eradicated by incubation with PJ-34 applied only once 24 hours after seeding (Figures 2 and 3). An efficient eradication of MCF-7 cells was observed after 48 hours of incubation with 10 μM PJ-34, whereas MDA231 cells were massively eradicated only after 72 hours of incubation with PJ-34, 20-30 μ*M*. Quiescent cells, brain cortical neurons, and cardiomyocytes were not impaired by incubation with the examined phenanthridine-derived PARP inhibitors (10 to 20 μM PJ-34, 100 μM Tiq-A, and 50 μM Phen [3,10,16,17]).

### PJ-34 prevented the development of MCF-7 and MDA231 xenotransplants in nude female mice

**Figure 6:**
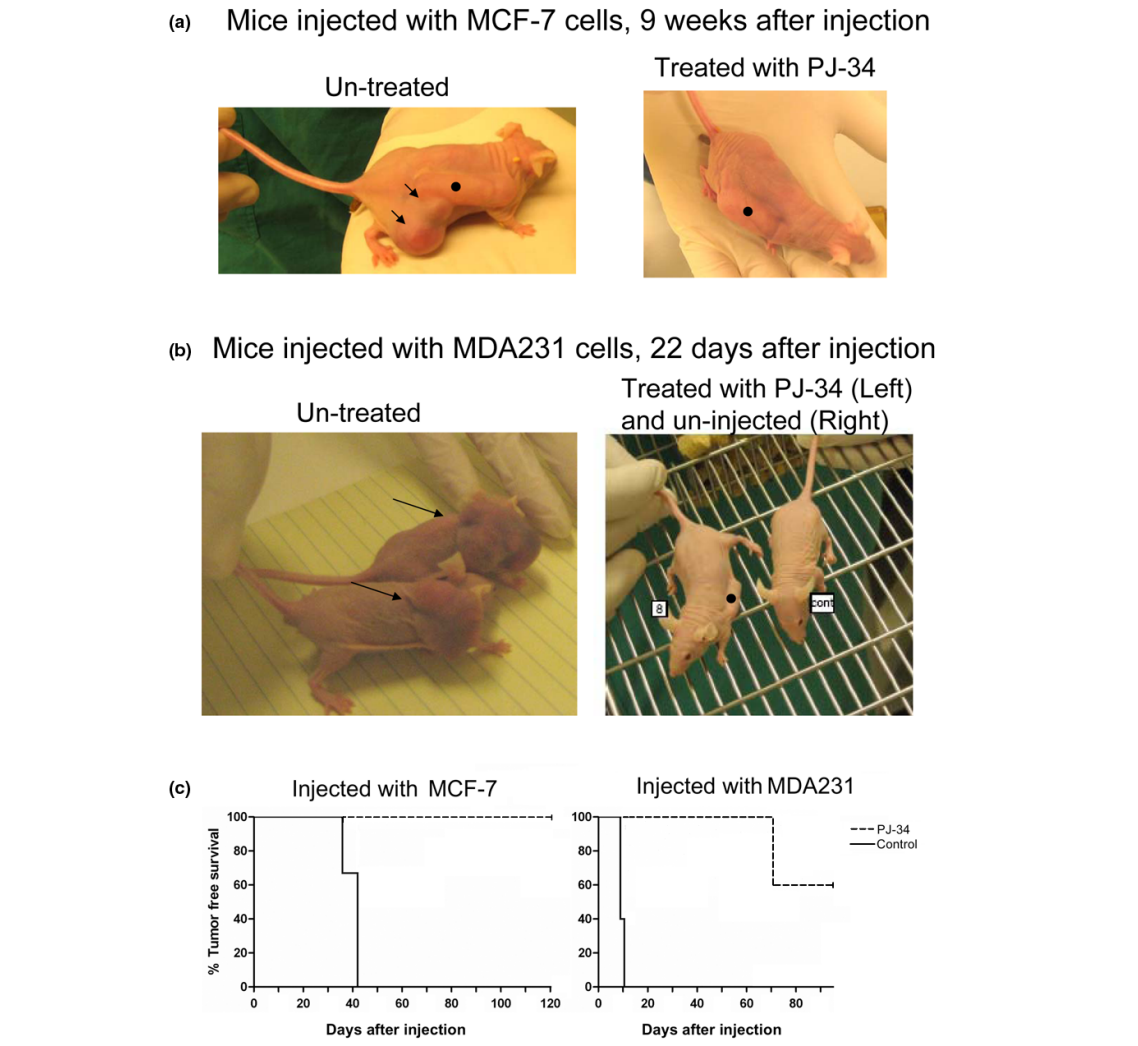
Treatment with PJ-34 prevented the development of MCF-7 and MDA231 xenotransplants. **(a) **Xenotransplants of MCF-7 developed within 6 to 7 weeks in three mice that were not treated with PJ-34 (control mice; tumors are indicated by *arrows*; **left**). Tumors did not develop in any of the three mice treated with PJ-34 for 14 days by a slow release of PJ-34 from a subcutaneously implanted osmotic pump (Alzet; pump •; Methods) (**Right**). Tumors were not detected during 4 months after injection with the MCF-7 cells. Each female CD-1 nu/nu mouse was injected with about 10^7 ^MCF-7 cells collected from 80% to 90% confluent cell cultures. Cells were immersed in Matrigel/PBS (Methods). Cells were injected near the pump, one hour after pump implantation. **(b) **Each CD-1 nu/nu female mouse was injected with about 10^7 ^MDA231 cells collected from 80% to 90% confluent cell cultures and immersed in Matrigel/PBS (Methods). Cells were injected near the pump, 24 hours after pump implantation. Xenotransplants of MDA231 developed within 10 days in five female mice that were not treated with PJ-34 (untreated mice). Tumors did not develop in five female mice treated for 14 days with a slow release from a subcutaneously implanted osmotic pump (Alzet pump). Tumors were not detected in these mice during 10 weeks after injection with MDA231 cells and 8 weeks after the treatment with PJ-34. A detailed presentation of this experiment is included in Additional data file 1. **(c) **Kaplan-Meier survival analysis is used for presenting tumor-free survival curves of mice injected with MCF-7 cells and mice injected with MDA231 cells, without or after treatment with PJ-34, as described earlier. The significance (log-rank significance test) was *P *= 0.0253 for mice injected with MCF-7 cells, and *P *= 0.0023 for mice injected with MDA231 cells.

*In vivo *experiments were carried out in nude female mice (nu/nu) injected subcutaneously with MCF-7 or MDA231 cells (Figure 6 and Additional data file 1). To test the effect of PJ-34 on the development of xenotransplants in the injected mice, PJ-34 (2 m*M *dissolved in 100 μl PBS) was inserted into subcutaneously implanted osmotic pumps (Alzet) that enable its constant slow release for 14 days (Methods). In the control nude mice, pumps contained only PBS, or pumps were not implanted. On the next day, 24 hours after pump implantation, each mouse was injected subcutaneously with approximately 10^7 ^MCF-7 or MDA231 cells dispersed in Matrigel (Methods). Tumors developed within 6 to 7 weeks in the control mice injected with MCF-7 cells and within 10 days in the control mice injected with MDA231 cells. One mouse died 3 weeks after being injected with MDA231 cells. In contrast, no visible tumors developed in the PJ-34-treated mice during 4 months after injection of MCF-7 cells and during the 10 weeks after injection with MDA231 cells (Figure 6a and 6b and Additional data file 1). Importantly, the 14-day treatment with a slow release of PJ-34 did not affect the vitality, growth, development, or any other behavior of the treated mice during the follow-up periods.

After 10 weeks, we detected tumors in two of the five mice that were injected with MDA231 cells and treated with PJ-34. These tumors were of human origin, as indicated by histochemistry (labeling with mouse anti-human mitochondria antibody (Millipore/Biotest) applied after blocking ("mouse-on-mouse"; Vector Labs/Zotal)).

Tumor-free survival curves [18] for mice injected with MCF-7 cells and for mice injected with MDA231 cells are presented in Figure 6c. The effect of treatment with PJ-34 on tumor-free survival is indicated, and significance was calculated with the log-rank significance test [18].

## Discussion

Findings indicating that polyADP-ribosylation is required for spindle formation [19] are in accordance with the observed G_2_/M arrest in both normal and malignant dividing cells treated with the potent PARP inhibitor PJ-34 (Figures 3, 4b, 5b). It is puzzling how normal cells overcome the G_2 _arrest while malignant cells die.

G_2 _arrest could be induced by DNA damage in proliferating cells [20]. Because of their genomic instability and fast proliferation, malignant cells might be more susceptible to inhibition of PARP mediated DNA repair than are normal proliferating cells [13,14]. However, eradication of MCF-7 and MDA231 cells was not shared by other non- phenanthridine-derived PARP inhibitors, and the examined cell lines MCF-7 and MDA231 were neither *BRCA-*deficient cells [21] nor *PTEN *(phosphatase and tensin homologue)-deficient cells [22], in which DNA-repair mechanisms are impaired [15,23].

Other possible mechanisms underlying G_2_/M arrest implicate signal-transduction pathways involving p53, p21/WAF1, cdc25c, cdc2, and suppressed cyclin-B gene expression [20,24-26]. Stabilization of p21 could promote G_2 _arrest and induce cell death [25]. ERK activation also is implicated in G_2_/M transition control. Nuclear translocation of cyclin B/Cdc2 complex, which is required for G_2_/M transition, could be mediated by ERK activation [20]. Other mechanisms involving ERK activation in G_2_/M transition control are not fully resolved. They include autostabilization of the p21cip/cyclin D1 complex leading to cell-cycle arrest, and mechanisms promoting degradation of cyclin-dependent inhibitors such as p27kip1 [26-31]. Various impairments in some of these regulatory mechanisms were detected in human cancer cell types.

Numerous primary human tumors and derived cell lines display a constitutive activation of ERK [8,31-34]. Some of them harbor mutations of the Ras protein that render it and the Raf-MEK-ERK cascade constitutively active [35-37]. Constitutively activated Raf has been found in many human cancers [35-37]. ERK targets are constitutively activated in B-Raf melanomas, colon cancer, PC-3 prostate cancer, and pancreatic cancer cells [37-39]. Growth and proliferation of the human breast cancer MCF-7 and MDA231 cells are also highly dependent on ERK activity [40-42].

Blocking the activity of ERK by blocking the Ras/Raf/MEK/ERK pathway is one of the main targets for human cancer treatment. However, previous clinical studies showed an insufficient antitumor activity of MEK inhibitors [39], suggesting that the MEK/ERK phosphorylation pathway is resilient to diminution of the activity of MEK and can adapt rapidly to maintain nearly normal ERK activation [39].

The recently disclosed augmentation of ERK activity in the nucleus by polyADP-ribosylated PARP-1 [3], and the fact that PARP-1 silencing with targeted siRNA downregulated ERK phosphorylation in nuclei of both MCF-7 and MDA231 cells (not shown), urged us to examine PARP inhibitors for their possible effect on cell proliferation in the absence of DNA-damaging factors. However, whereas phenanthridine-derived PARP inhibitors efficiently arrested the cell proliferation of MCF-7 and MDA231 cancer cells, other potent PARP inhibitors did not cause a similar effect. PARP inhibitor, ABT-888 (benzimidazole-4-carboxamide) hardly affected MCF-7 and MDA231 cells (not shown), and potent PARP inhibitors efficiently eradicating *BRCA *mutated breast cancer cells did not similarly eradicate MCF-7 cells [13,14] and cancer cells not carrying *BRCA *mutations [43]. Hence, additional features of the phenanthridine-derived PARP inhibitors apparently underlie their very promising potency to arrest proliferation and cause cell death in cancer cells lacking mutations that impair DNA repair, without impairing normal proliferating or quiescent cells.

## Conclusions

Phenanthridine-derived PARP inhibitors possess a promising potency to arrest proliferation (G_2_/M arrest) and cause cell death in MCF-7 and MDA231 human breast cancer cells, without impairing normal proliferating human epithelial cells, fibroblasts, or quiescent cells (neurons, cardiomyocytes).

## Abbreviations

ERK: externally regulated kinase; MEK: mitogen-activated protein kinase kinase; PARP: polyADP-ribose polymerase; PTEN: phosphatase and tensin homologue; Raf: mitogen-activated protein kinase kinase kinase.

## Competing interests

The authors declare that they have no competing interests. This study was not supported by any company or commercial fund.

MC-A is the inventor of patent WO 2009/0477052, submitted by Tel-Aviv University.

## Authors' contributions

Corresponding author MC-A designed the experiments, analyzed the data, and wrote the manuscript. DI-R, LV, AC, SI, and DC performed the experiments. DF reviewed the manuscript and supplied mouse embryonic fibroblasts.

## Supplementary Material

Additional file 1 The following additional data are available with the online version of this article: A documented experiment, testing PJ-34 treatment in nude mice injected with MDA231 breast cancer cells (Additional data file 1). Click here for file

## References

[B1] Schreiber V Dantzer FJ-C Ame de Murcia G PolyADP-ribose: novel functions for an old molecule Nat Rev Mol Cell Biol 2006 7 517 528 10.1038/nrm1963 16829982

[B2] Kraus WL Transcriptional control by PARP-1:chromatin modulation, enhancer-binding, coregulation, and insulation Curr Opin Cell Biol 2008 20 294 302 18450439 10.1016/j.ceb.2008.03.006 PMC2518631

[B3] Cohen-Armon M Visochek L Rozensal D Kalal A Geistrikh I Klein R Bendetz-Nezer S Yao Z Seger R DNA-independent PARP-1 activation by phosphorylated ERK2increases Elk1 activity: a link to histone acetylation Mol Cell 2007 25 297 308 10.1016/j.molcel.2006.12.012 17244536

[B4] Cohen-Armon M PARP-1 activation in the ERK signaling pathway Trends Pharmacol Sci 2007 28 556 560 10.1016/j.tips.2007.08.005 17950909

[B5] Cohen-Armon M PARP-1 activation mediates the expression of immediate early genes implicated in long-term memory formation FEBS J 2008 275 Suppl 1 92

[B6] Carbone M Rossi MN Cavaldesi M Notari A Amati P Maione R Poly(ADP-ribosyl)ation is implicated in the G0-G1 transition of resting cells Oncogene 2008 27 6083 6092 10.1038/onc.2008.221 18663363

[B7] Buchwalter G Gross C Wasylyk B Ets ternary complex transcription factors Gene 2004 324 1 14 10.1016/j.gene.2003.09.028 14693367

[B8] Hoshino R Chantani Y Yamori T Tsuruo T Oka H Yoshida O Shimada Y Ari-i S Wada H Fujimoto J Kohno M Constitutive activation of the 41/42-kDa mitogen-activated protein kinase signaling pathway in human tumors Oncogene 1999 18 813 822 10.1038/sj.onc.1202367 9989833

[B9] Ratnam K Low JA Current development of clinical inhibitors of polyADP-ribose polymerase in oncology Clin Cancer Res 2007 13 1383 1388 10.1158/1078-0432.CCR-06-2260 17332279

[B10] Chiarugi A Meli E Calvani M Picca R Picca R Baronti R Camaioni E Costantino G Marinozzi M Pellegrini-Giampietro DE Pellicciari R Moroni F Novel isoquinolinone-derived inhibitors of polyADP-ribose polymerase-1: pharmacological characterization and neuroprotective effects in an *in vitro *model of cerebral ischemia J Pharmacol Exp Ther 2003 305 943 949 10.1124/jpet.103.048934 12606624

[B11] Peralta-Leal A Rodríguez-Vargas JM Aguilar-Quesada R Rodríguez MI Linares JL de Almodóvar MR Oliver JF PARP inhibitors: new partners in the therapy of cancer and inflammatory diseases Free Radic Biol Med 2009 47 13 26 10.1016/j.freeradbiomed.2009.04.008 19362586

[B12] Jagtap P Szabó C Poly(ADP-ribose) polymerase and the therapeutic effects of its inhibitors Nat Rev Drug Disc 2005 4 421 440 10.1038/nrd1718 15864271

[B13] Bryant HE Schultz N Thomas HD Parker KM Flower D Lopez E Kyle S Meuth M Curtin NJ Helleday T Specific killing of BRCA2-deficient tumours with inhibitors of poly(ADP-ribose) polymerase Nature 2005 434 913 917 10.1038/nature03443 15829966

[B14] Farmer H McCabe N Lord CJ Tutt ANJ Johnson DA Richardson TB Santarosa M Dillon KJ Hickson I Knights C Martin NM Jackson SP Smith GC Ashworth A Targeting the DNA repair defect in BRCA mutant cells as a therapeutic strategy Nature 2005 434 917 921 10.1038/nature03445 15829967

[B15] Grant SG Das R Cerceo CM Rubinstein WS Latimer JJ Elevated levels of somatic mutation in a manifesting BRCA1 mutation carrier Pathol Oncol Res 2007 13 276 283 10.1007/BF02940305 18158561 PMC4301730

[B16] Cohen-Armon M Visochek L Katzoff A Levitan D Susswein AJ Klein R Valbrun M Schwartz JH Long-term memory requires polyADP-ribosylation Science 2004 304 1820 1823 10.1126/science.1096775 15205535

[B17] Visochek L Vulih I Steingart RA Klein R Priel E Gozes I Cohen-Armon M PolyADP-ribosylation is involved in neurotrophic activity J Neurosci 2005 25 7420 7428 10.1523/JNEUROSCI.0333-05.2005 16093393 PMC6725295

[B18] Harrington D Linear rank tests in survival analysis Encyclopedia of biostatistics 2005 New York: Wiley Interscience DOI: 10.1002/0470011815.b2a11047

[B19] Chang P Jacobson MK Mitchison TJ Poly(ADP-ribose)is required for spindle assembly and structure Nature 2004 432 645 649 10.1038/nature03061 15577915

[B20] Stark GR Taylor WR Control of the G2/M transition Mol Biotechnol 2006 32 227 248 10.1385/MB:32:3:227 16632889

[B21] Fustier P Le Corre L Chalabi N Vissac-Sabatier C Communal Y Bignon Y-J Bernard-Gallon DJ Resveratrol increases BRCA1 and BRCA2 mRNA expression in breast tumour cell lines Br J Cancer 2003 89 168 172 12838319 10.1038/sj.bjc.6600983 PMC2394230

[B22] Yu K Toral-Barza L Discafani C Zhang W-G Skotnicki J Frost P Gibbons JJ mTOR, a novel target in breast cancer: the effect of CCI-779, an mTOR inhibitor, in preclinical models of breast cancer Endocr Relat Cancer 2001 8 249 258 10.1677/erc.0.0080249 11566616

[B23] Mendes-Pereira AM Martin SA Brough R McCarthy A Taylor JR Kim J-S Waldman T Lord CJ Ashworth A Synthetic lethal targeting of *PTEN* mutant cells with PARP inhibitors EMBO Mol Med 2009 1 315 322 20049735 10.1002/emmm.200900041 PMC3378149

[B24] Yoshida M Matsui Y Iizuka A Ikarashi Y G2-phase arrest through p21(WAF1/Cip1) induction and cdc2 repression by gnidimacrin in human hepatoma HLE cells Anticancer Res 2009 29 1349 1354 19414386

[B25] Abbas T Dutta A p21 in cancer: intricate networks and multiple Activities Nat Rev Cancer 2009 9 400 414 10.1038/nrc2657 19440234 PMC2722839

[B26] Nigam N Prasad S George J Shukla Y Lupeol induces p53 and cyclin-B-mediated G2/M arrest and targets apoptosis through activation of caspase in mouse skin Biochem Biophys Res Commun 2009 381 253 258 10.1016/j.bbrc.2009.02.033 19232320

[B27] Aaltonen K Amini R-M Heikkilä P Aittomäki K Tamminen A Nevanlinna H Blomqvist C High cyclin B1 expression is associated with poor survival in breast cancer Br J Cancer 2009 100 1055 1060 10.1038/sj.bjc.6604874 19293801 PMC2669992

[B28] Chambard J-C Lefloch R Pouyssegur J Lenormand P ERK implication in cell cycle regulation Biochim Biophys Acta 2007 1773 1299 1310 10.1016/j.bbamcr.2006.11.010 17188374

[B29] Whitmarsh AJ Davis RJ Transcription factor AP-1 regulation by mitogen-activated protein kinase signal transduction pathways J Mol Med 1996 74 589 607 10.1007/s001090050063 8912180

[B30] Shaulian E Karin M AP-1 in cell proliferation and survival Oncogene 2001 20 2390 2400 10.1038/sj.onc.1204383 11402335

[B31] Milde-Langosch K The Fos family of transcription factors and their role in tumourigenesis Eur J Cancer 2005 41 2449 2461 10.1016/j.ejca.2005.08.008 16199154

[B32] Brunet A Roux D Lenomand P Dowd S Keyse S Pouyssegur J Nuclear translocation of p42/p44 mitogen-activated protein kinase is required for growth factor-induced gene expression and cell cycle entry EMBO J 1999 18 664 674 9927426 10.1093/emboj/18.3.664 PMC1171159

[B33] Chalmers CJ Gilly R March HN Balmanno K Cook SJ The duration of ERK1/2 activity determines the activation of c-Fos and Fra-1 and the composition and quantitative transcriptional output of AP-1 Cell Signal 2007 19 695 704 10.1016/j.cellsig.2006.09.001 17052890

[B34] Balmanno K Cook SJ Sustained MAP kinase activation is required for the expression of cyclin D1, p21 and a subset of AP-1 proteins in CCL39 cells Oncogene 1999 18 3085 3097 10.1038/sj.onc.1202647 10340380

[B35] Davies H Bignell GR Cox C Stephens P Edkins S Clegg S Teague J Woffendin H Garnett MJ Bottomley W Davis N Dicks E Ewing R Floyd Y Gray K Hall S Hawes R Hughes J Kosmidou V Menzies A Mould C Parker A Stevens C Watt S Hooper S Wilson R Jayatilake H Gusterson BA Cooper C Shipley J Mutations of the B-RAF gene in human cancer Nature 2002 417 949 954 10.1038/nature00766 12068308

[B36] Karasarides M Chiloeches A Hayward R Niculescu-Duvaz D Scanlon I Friedlos F Ogilvie L Hedley D Martin J Marshall CJ Springer CJ Marais R B-RAF is a therapeutic target in melanoma Oncogene 2004 23 6292 6298 10.1038/sj.onc.1207785 15208680

[B37] Mercer KE Pritchard CA Raf proteins and cancer: B-Raf is identified as a mutational target Biochim Biophys Acta 2003 1653 25 40 12781369 10.1016/s0304-419x(03)00016-7

[B38] Tan X Egami H Abe M Nazawa F Hirota M Ogawa M Involvement of MMP-7 in invasion of pancreatic cancer cells through activation of the EGFR mediated MEK-ERK signal transduction pathway J Clin Pathol 2005 58 1242 1248 16311341 10.1136/jcp.2004.025338 PMC1770786

[B39] Rinehart J Adjei AA LoRusso PMD Hecht RJ Natale RB Hamid O Varterasian M Asbury P Kaldjian EP Gulyas S Mitchell DY Herrera R Sebolt-Leopold JS Meyer MB Multicenter phase II study of the oral MEK inhibitor, CI-1040, in patients with advanced non-small-cell lung, breast, colon, and pancreatic cancer J Clin Oncol 2004 22 4456 4462 10.1200/JCO.2004.01.185 15483017

[B40] Duan R Xie W Burghardt RC Safe S Estrogen receptor-mediated activation of the serum response element in MCF-7 cells through MAPK-dependent phosphorylation of Elk1 J Biol Chem 2001 276 11590 11598 10.1074/jbc.M005492200 11145955

[B41] Lu C Shen Q DuPre E Kim H Hilsenbeck S Brown PH cFos is critical for MCF-7 breast cancer cell growth Oncogene 2005 24 6516 6524 10.1038/sj.onc.1208661 16027729

[B42] Camirand A Zakikhani M Young F Pollak M Inhibition of insulin-like growth factor-1 receptor signaling enhances growth-inhibitory and proapoptotic effects of gefitinib (Iressa) in human breast cancer cells Breast Cancer Res 2005 7 R570 R579 15987464 10.1186/bcr1028 PMC1175059

[B43] Fong PC Boss DS Yap TA Tutt A Wu P Mergui-Roelvink M Mortimer P Swaisland H Lau A O'Connor MJ Ashworth A Carmichael J Kaye SB Schellens JH de Bono JS Inhibition of poly(ADP-ribose) polymerase in tumors from *BRCA *mutation carriers N Engl J Med 2009 361 123 134 10.1056/NEJMoa0900212 19553641

