# Supplementary material for: A selective eradication of human nonhereditary breast cancer cells by phenanthridine-derived polyADP-ribose polymerase inhibitors
Source: Breast Cancer Res. 2009 Nov 9;11(6):R78. doi: 10.1186/bcr2445 (PMC2815540; doi:10.1186/bcr2445)
Supplement: Additional file 1 — The following additional data are available with the online version of this article: A documented experiment, testing PJ-34 treatment in nude mice injected with MDA231 breast cancer cells (Additional data file 1). [file bcr2445-S1.pdf]

A documented experiment, testing PJ-34 treatment in nude mice injected with MDA231 cells

Slides 3-7: Mice treated with PJ-34, dripping from sub-cutan Implanted osmotic pump (•). Pumps were implanted 24 hours before injection with MDA231 cells.

Slide 8: untreated and un-injected mice (Control).

Slides 9-13: Mice untreated with PJ-34 developed tumors 10 days after injection with MDA231 cells

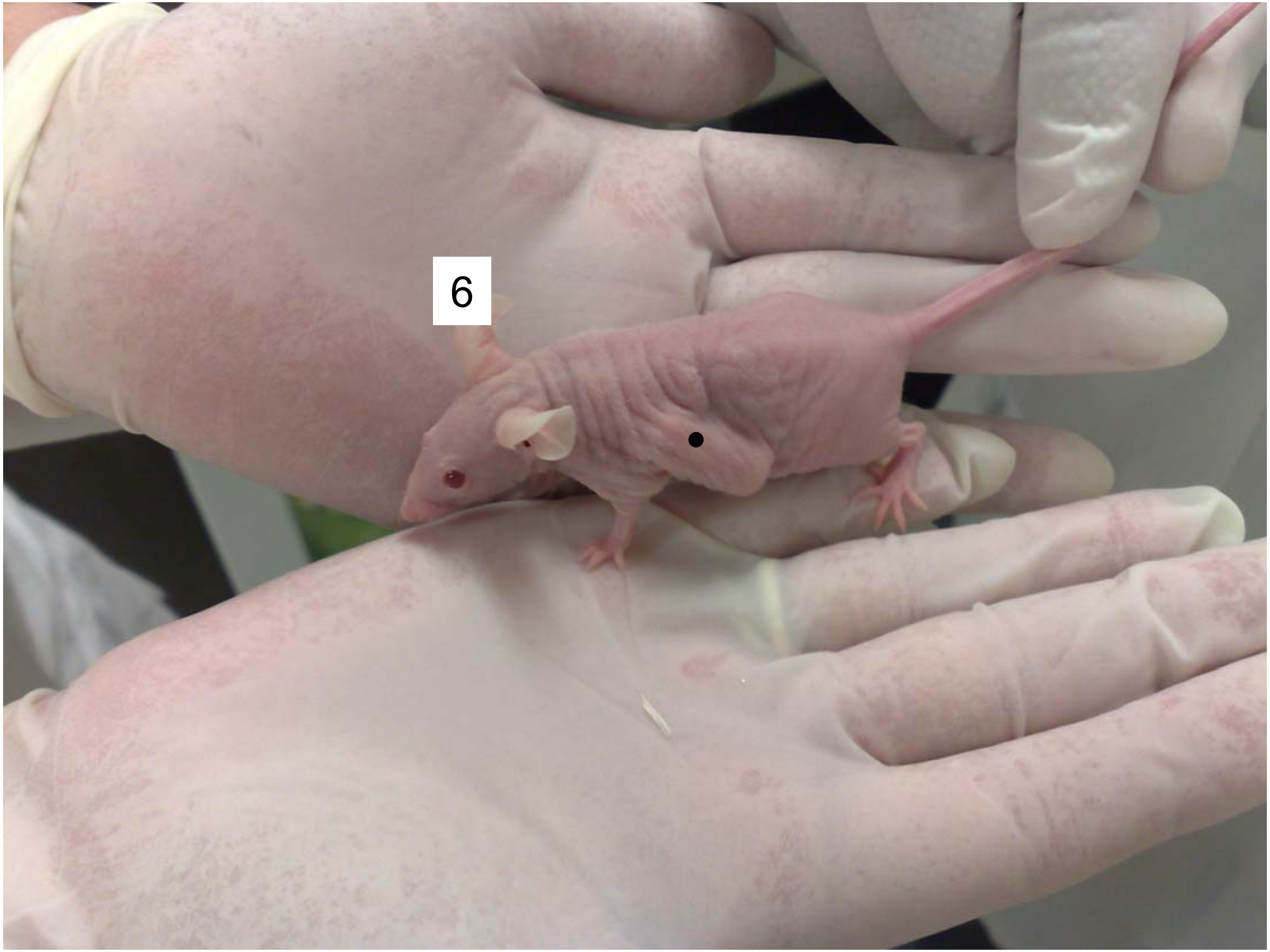
6

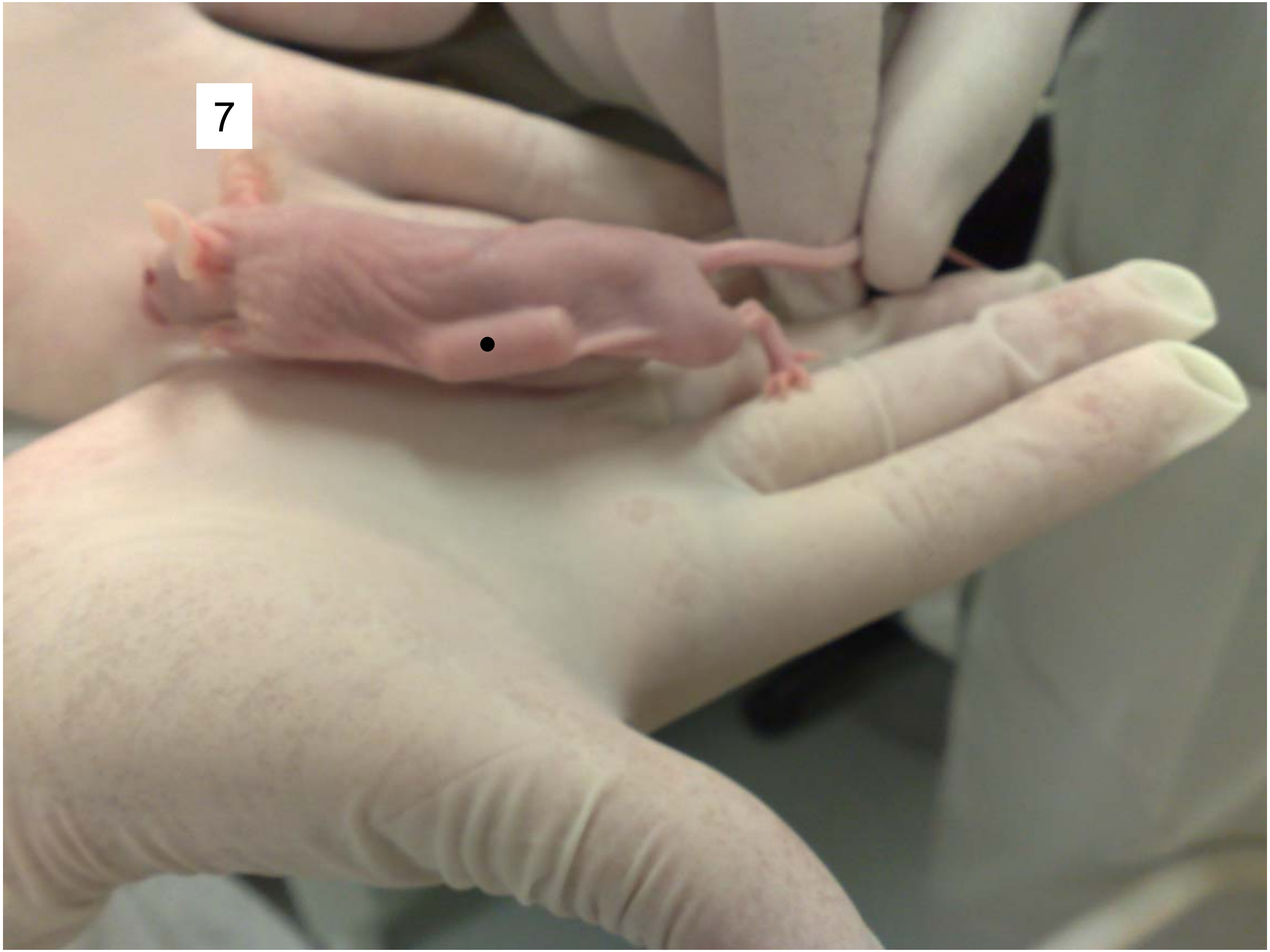
7

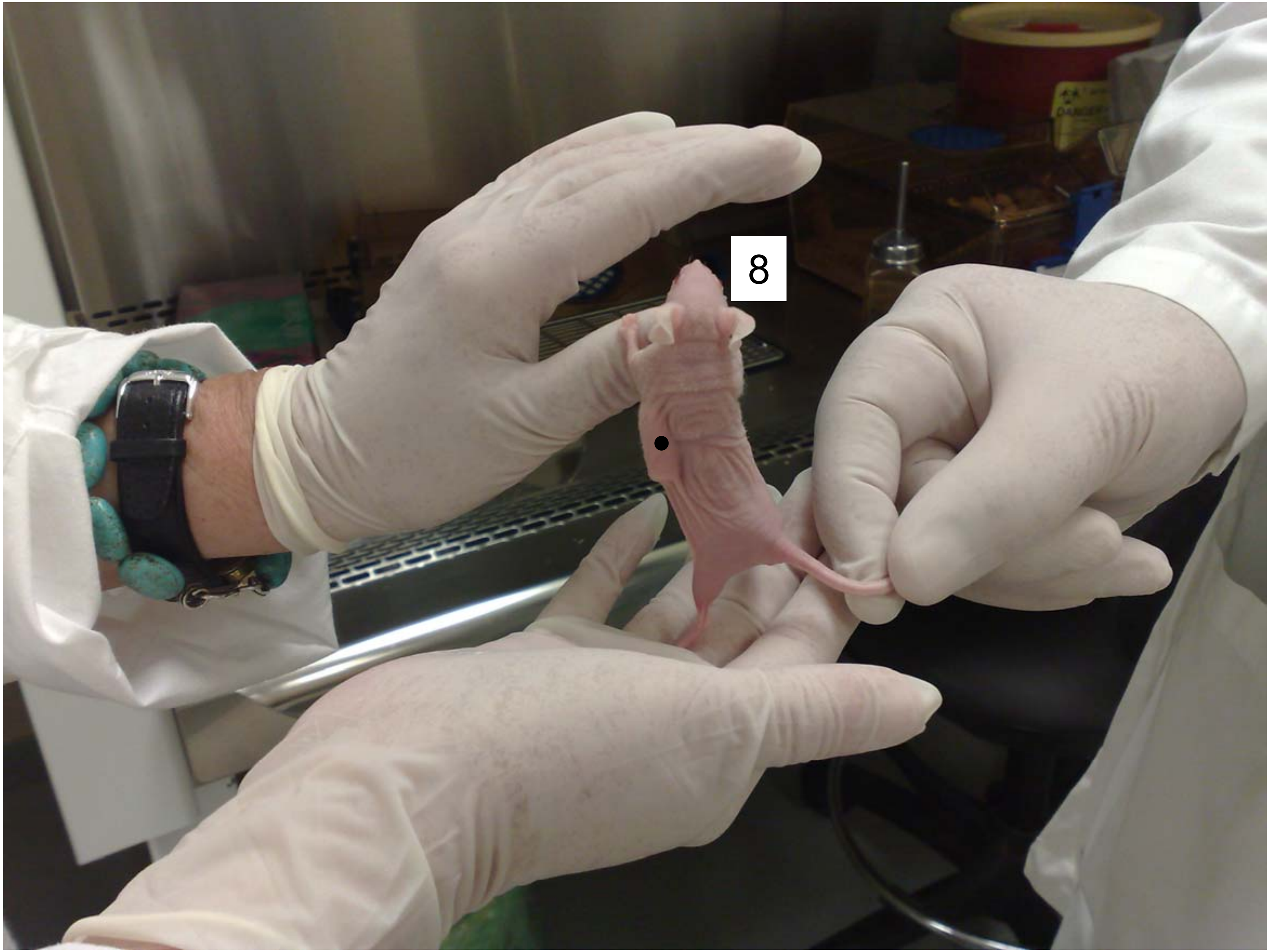
8

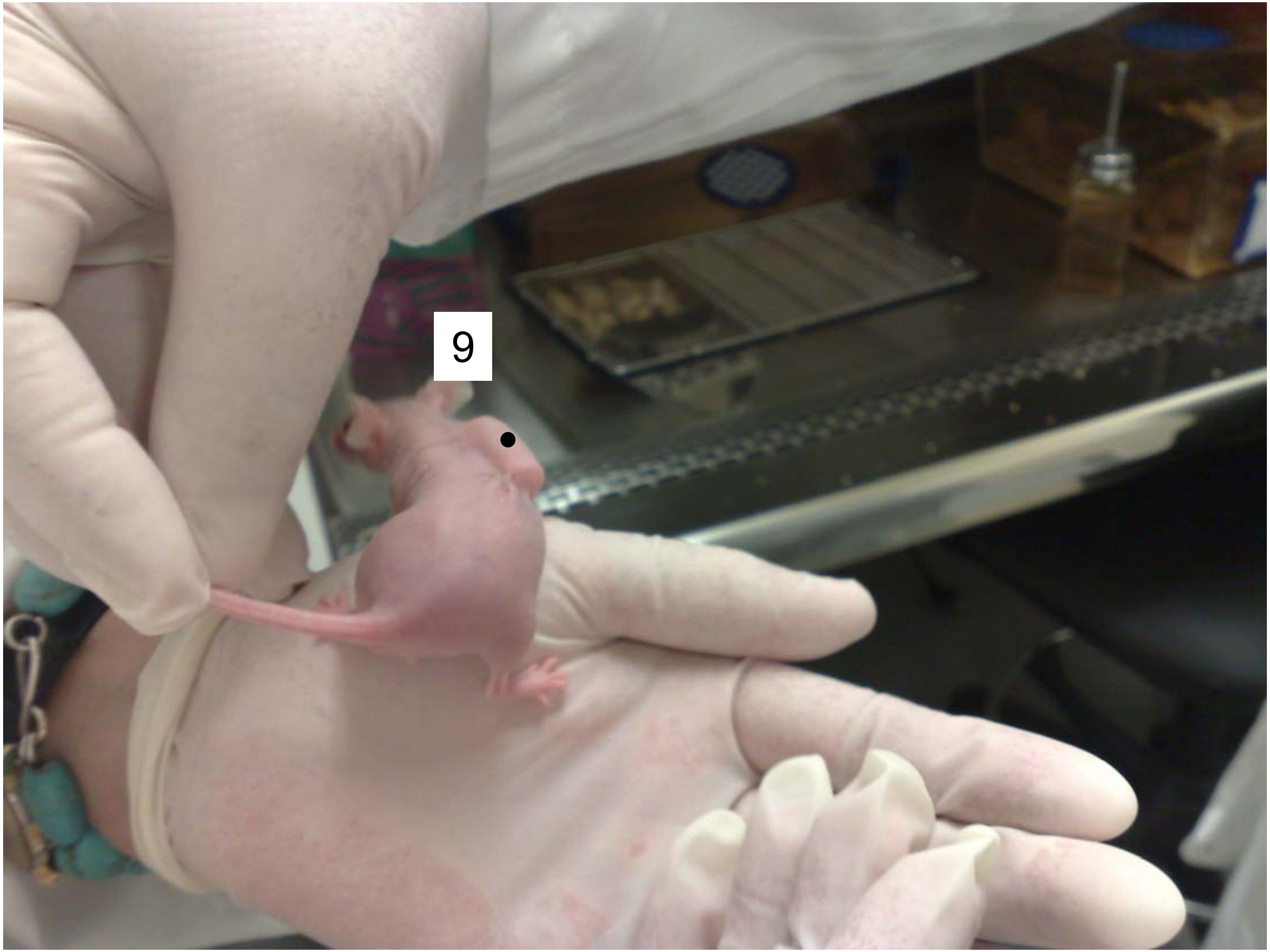
9

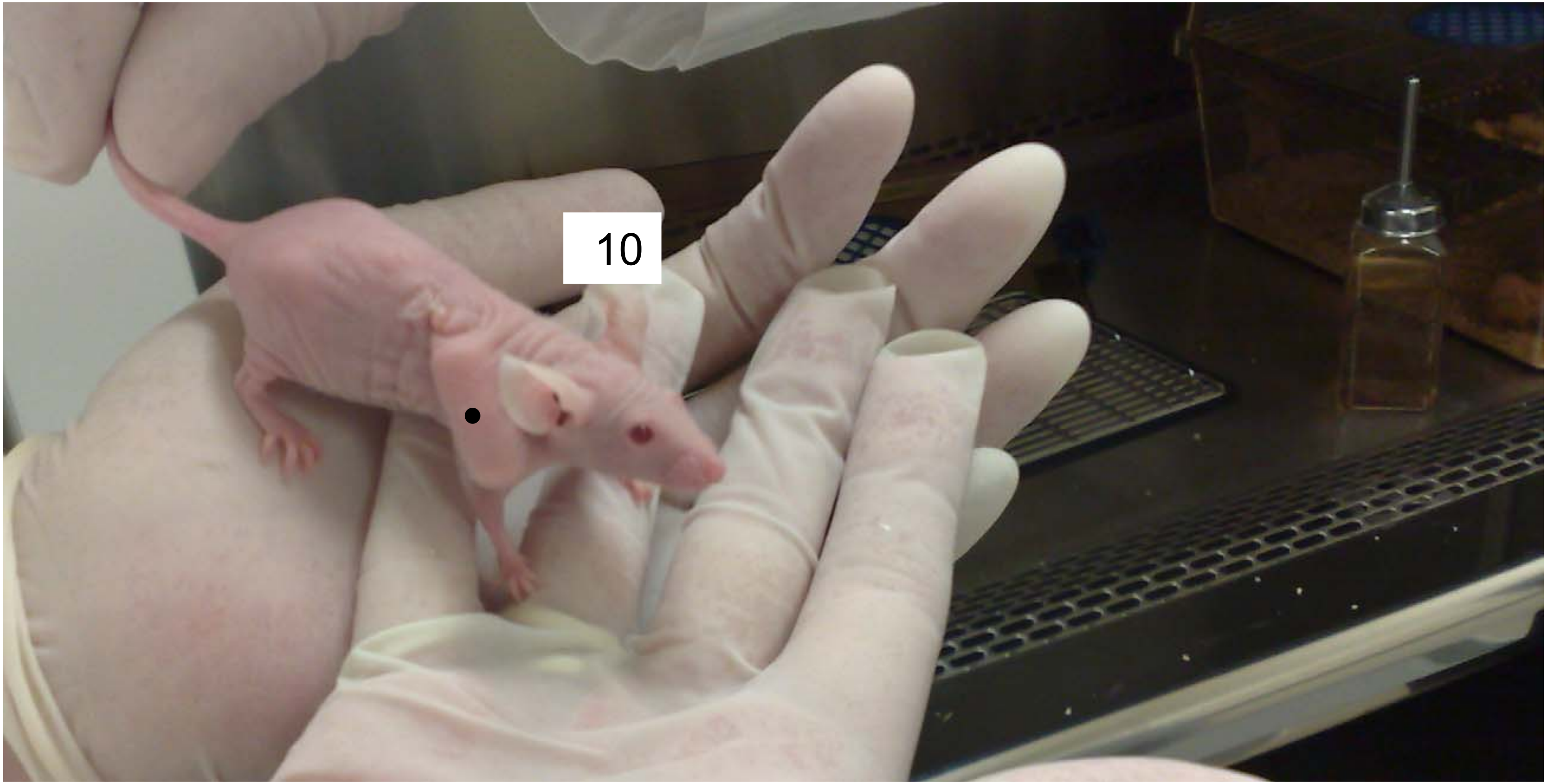
10

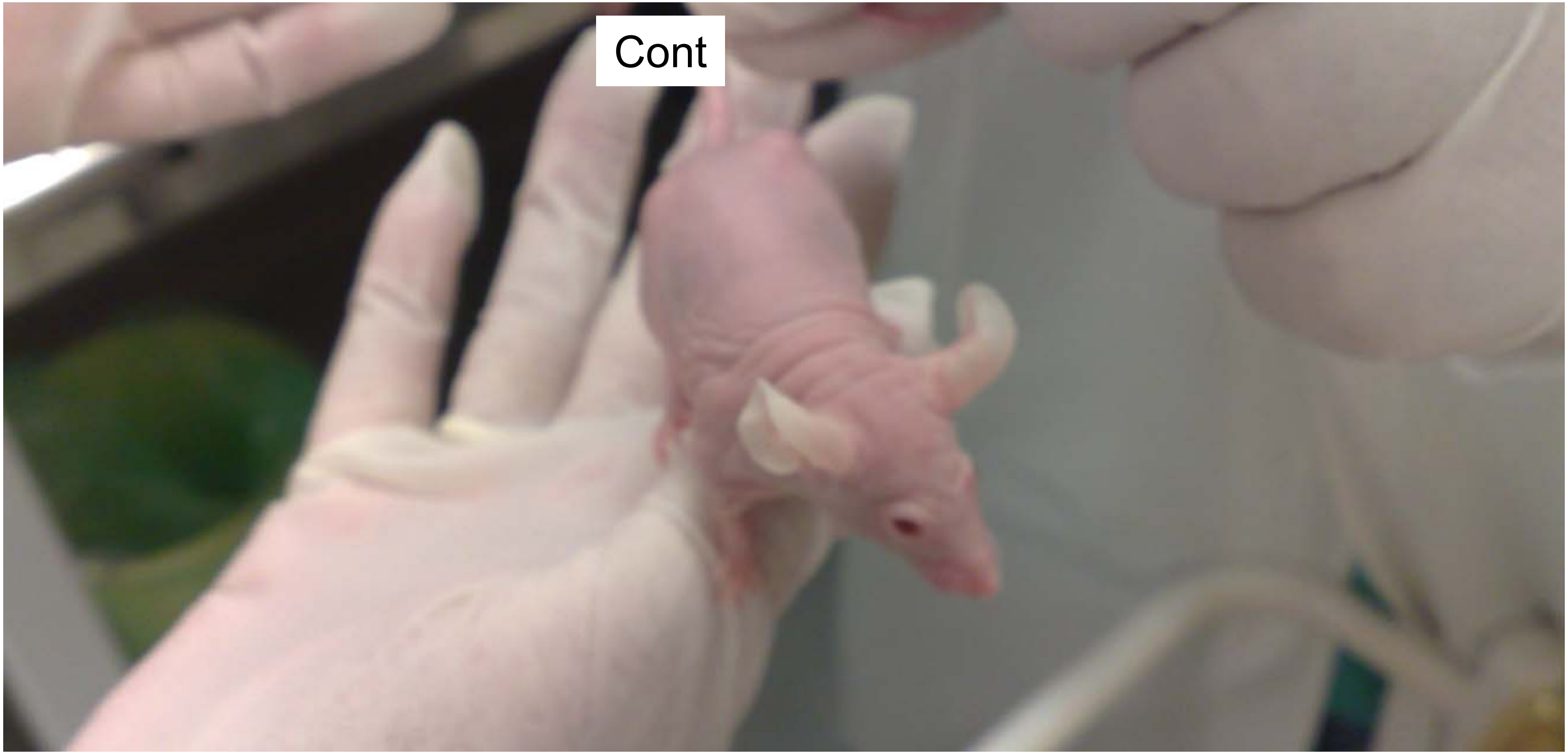
Cont

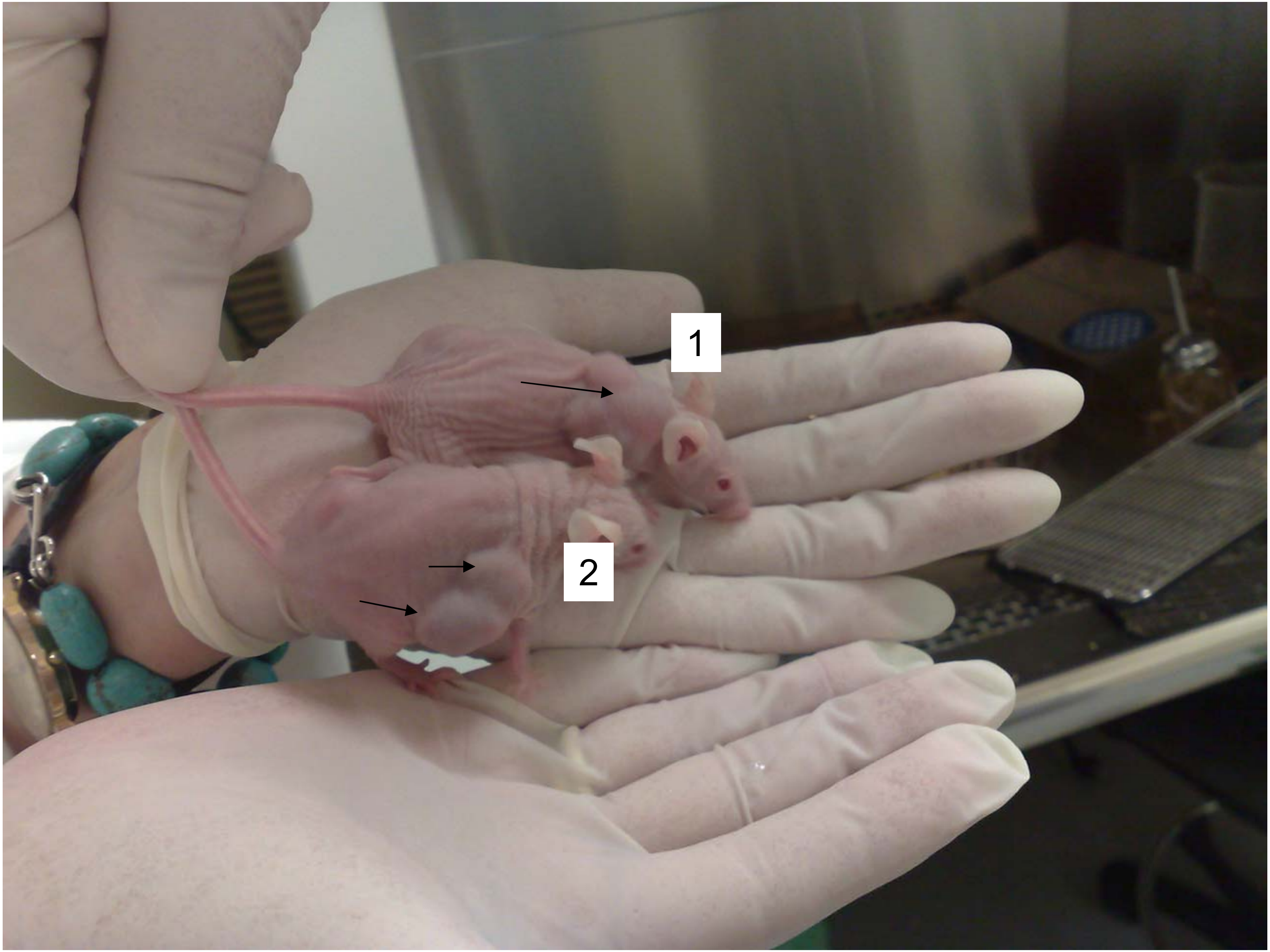
1
2

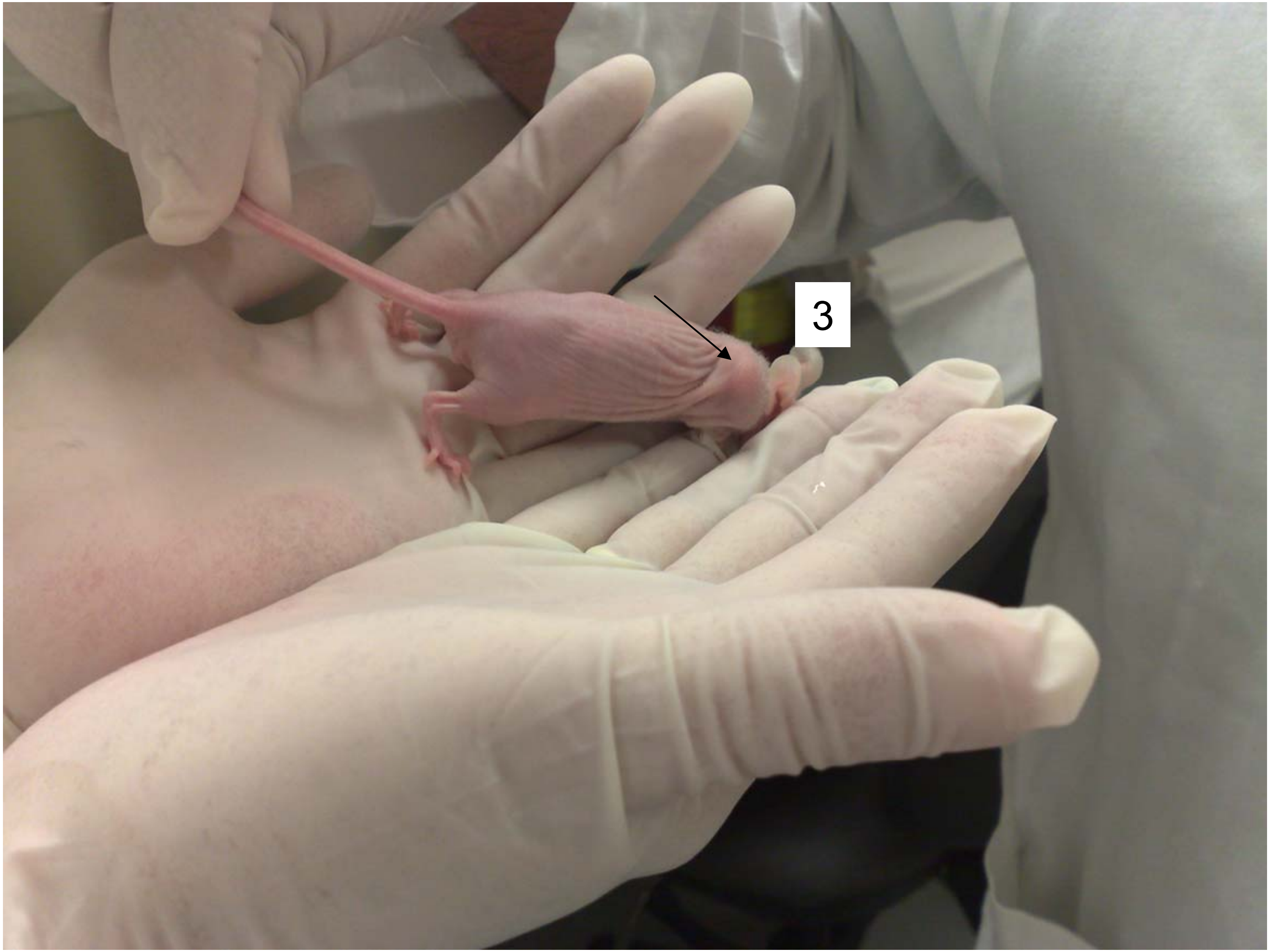
3

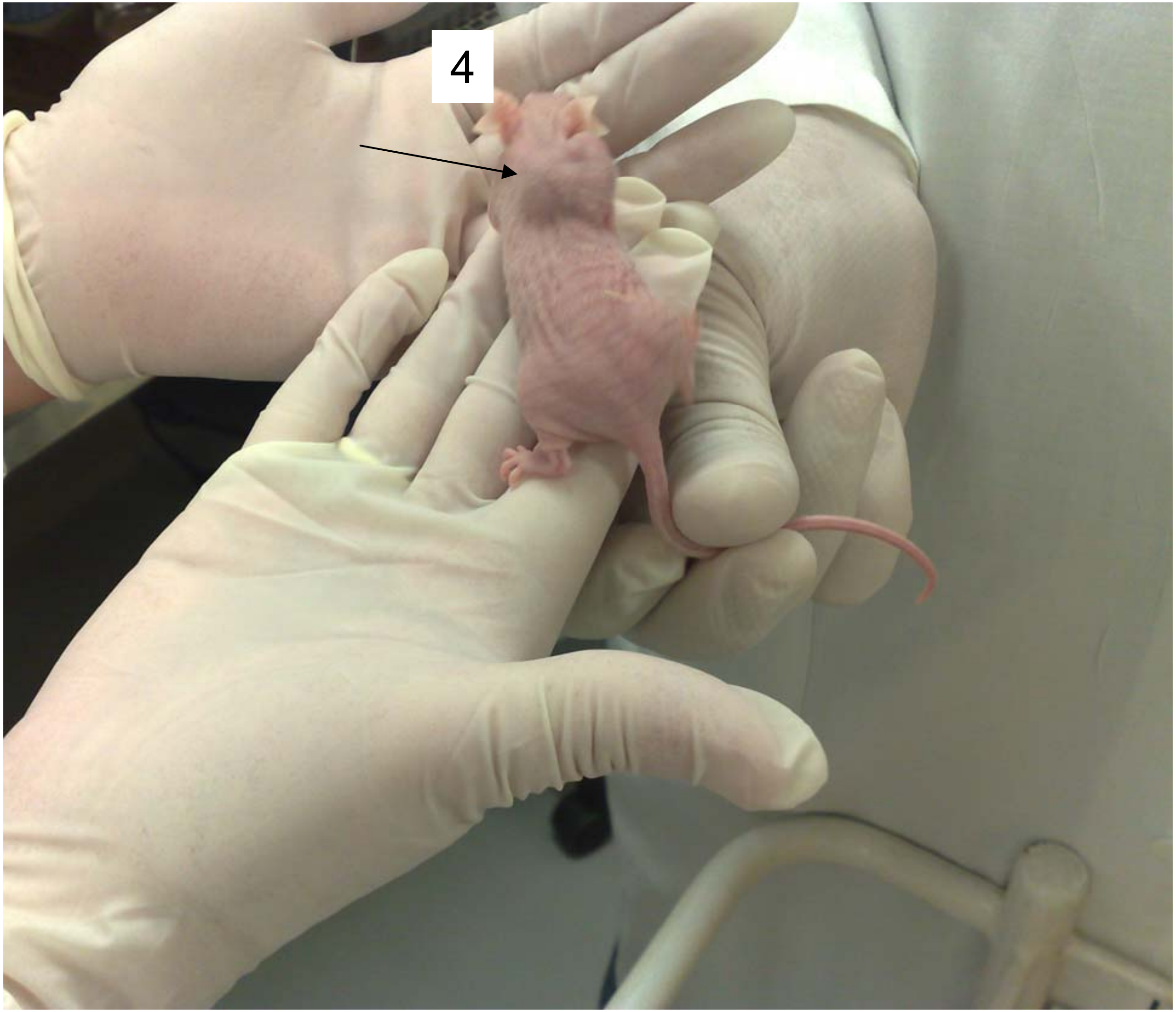
4

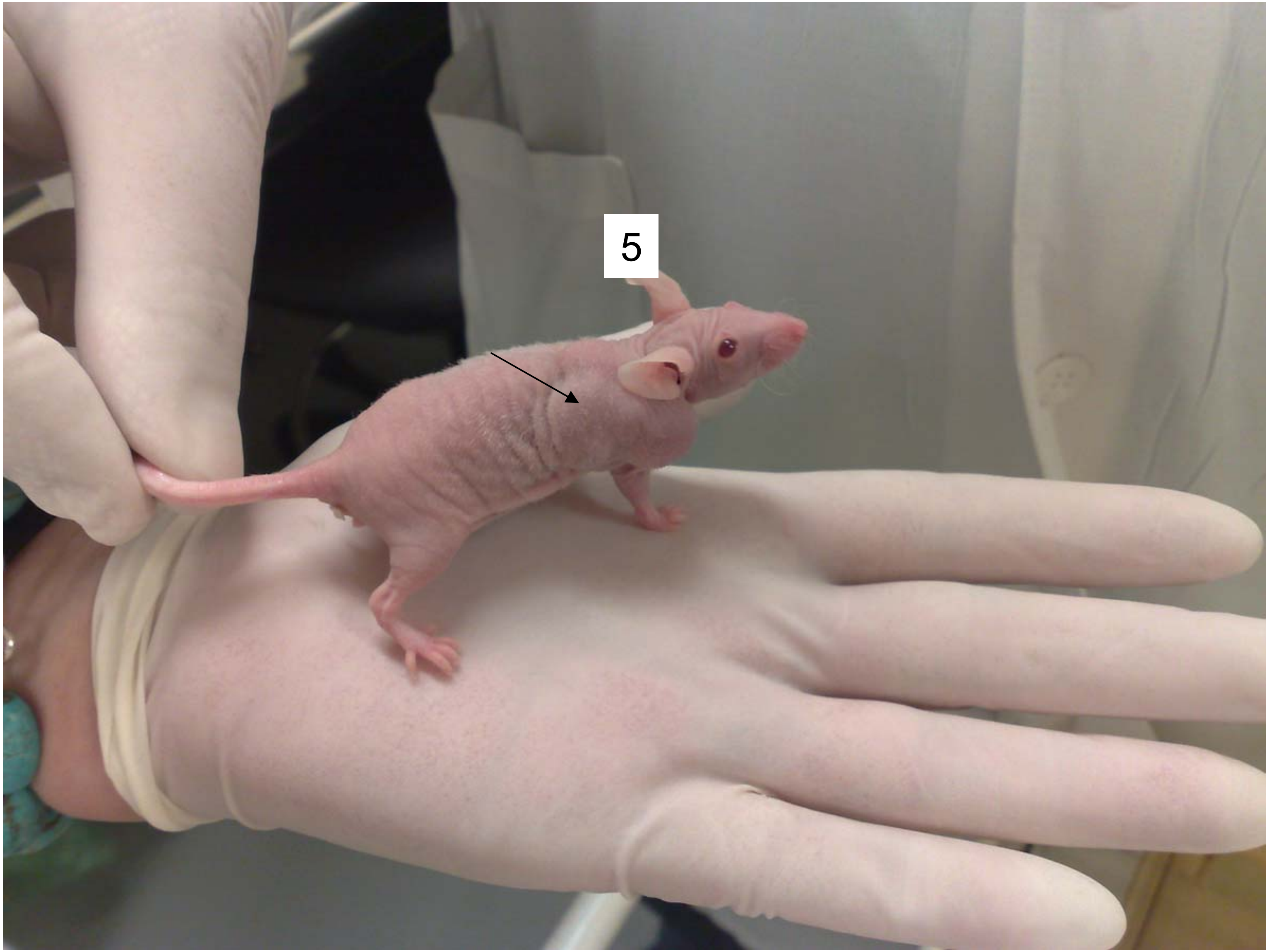
5

12 days later:

Slides 14-15: tumors developed in the untreated mice (# 1-5). One mouse died on day 22. The other 4 were photographed and sacrificed.

Slide 16: Mice treated with PJ-34 (# 6-10), 8 days after the treatment ceased. No tumors observed in any of them.

Control: untreated and un-injected mouse

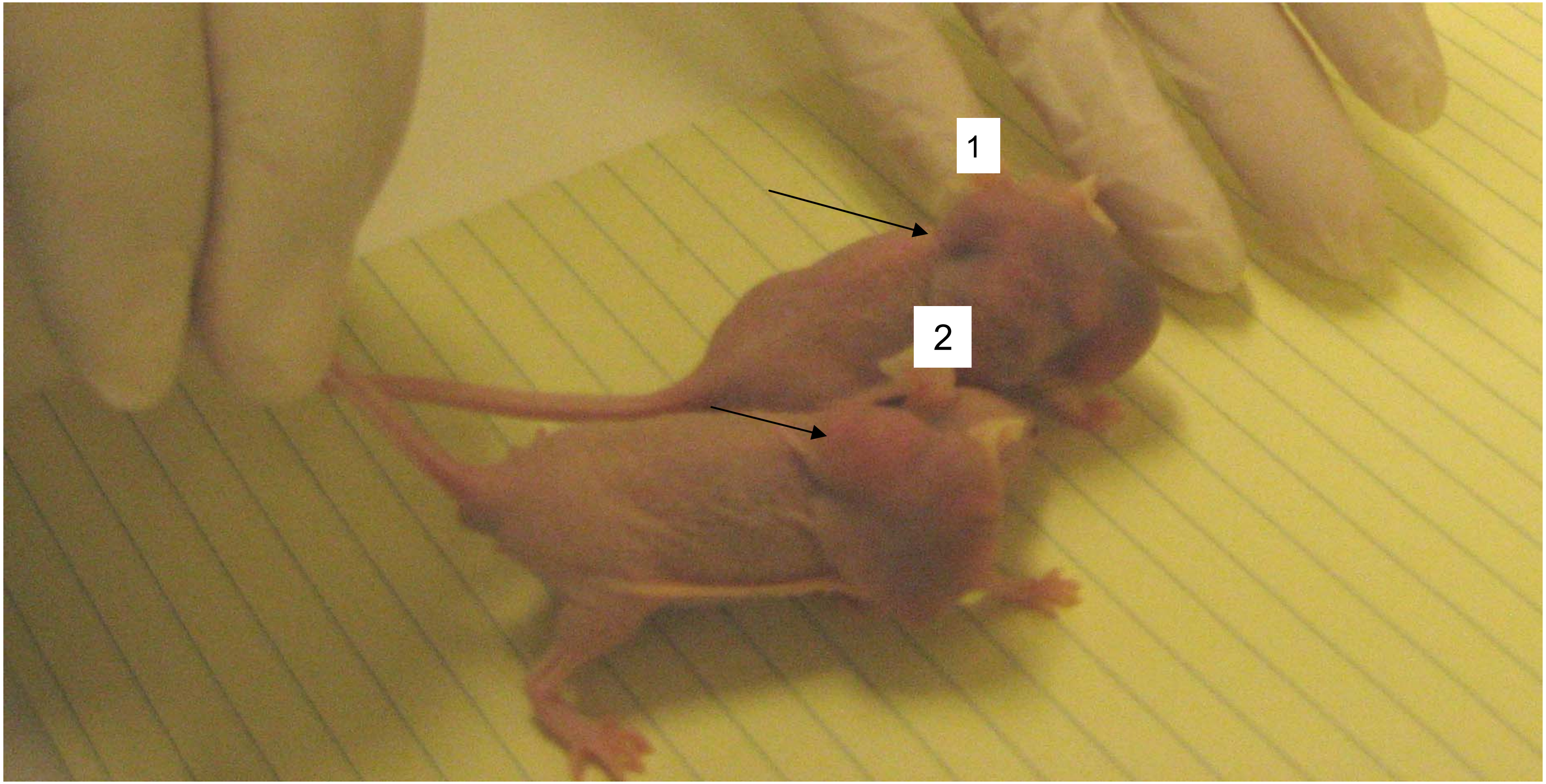
1
2

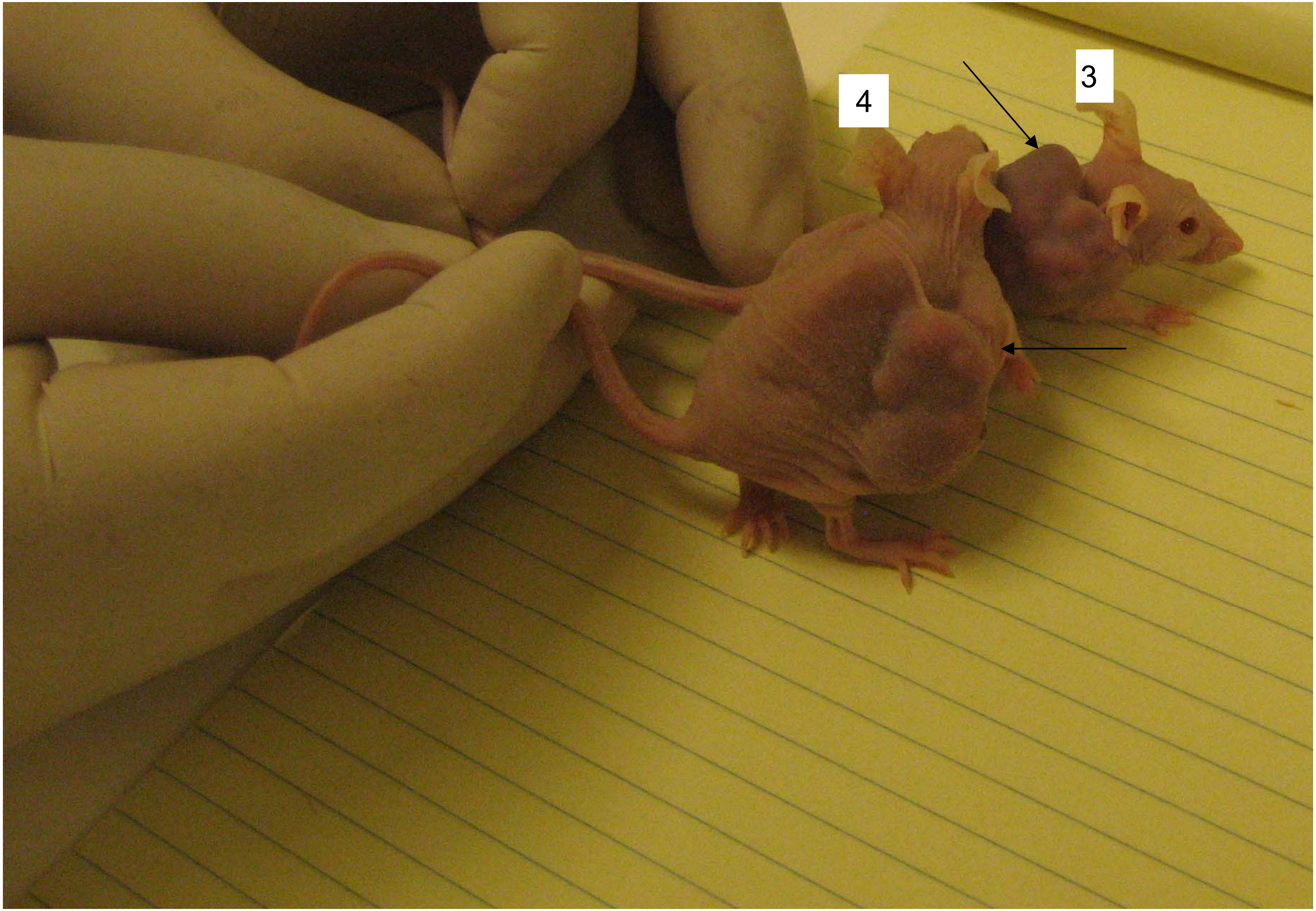
4
3

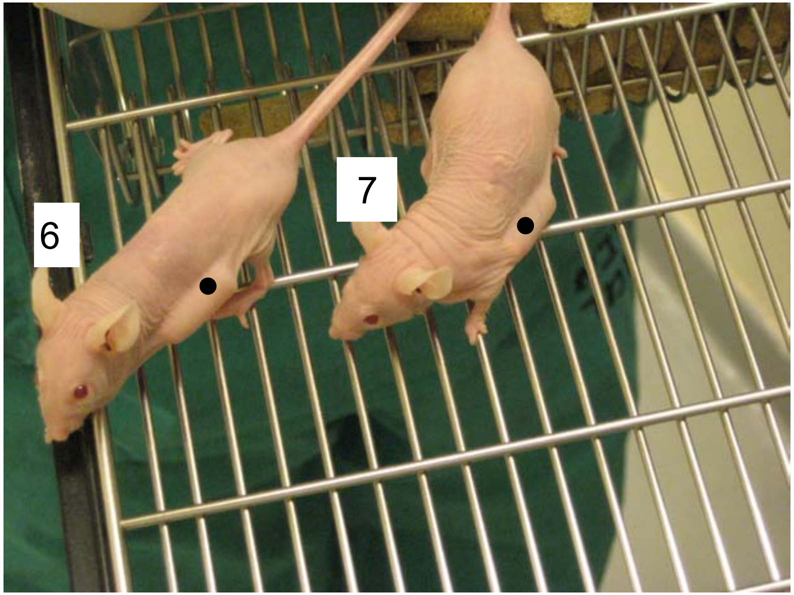
6
7

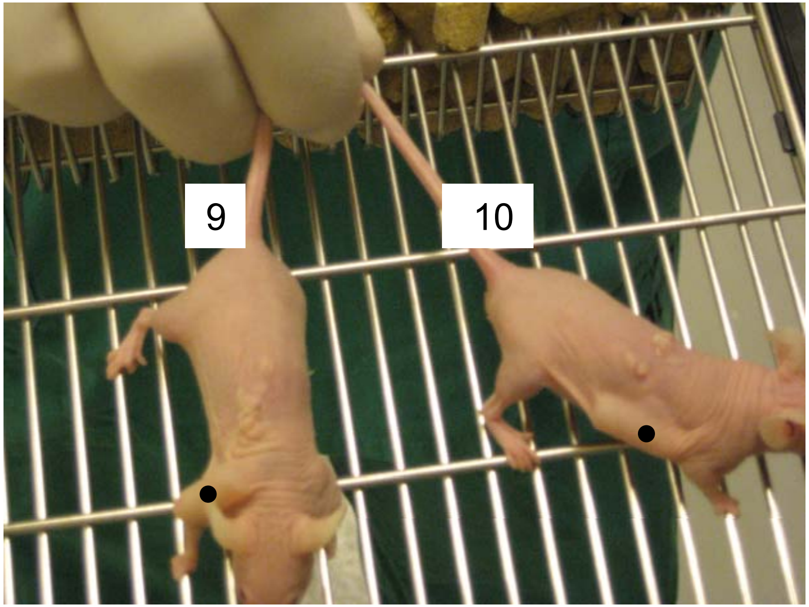
9
10

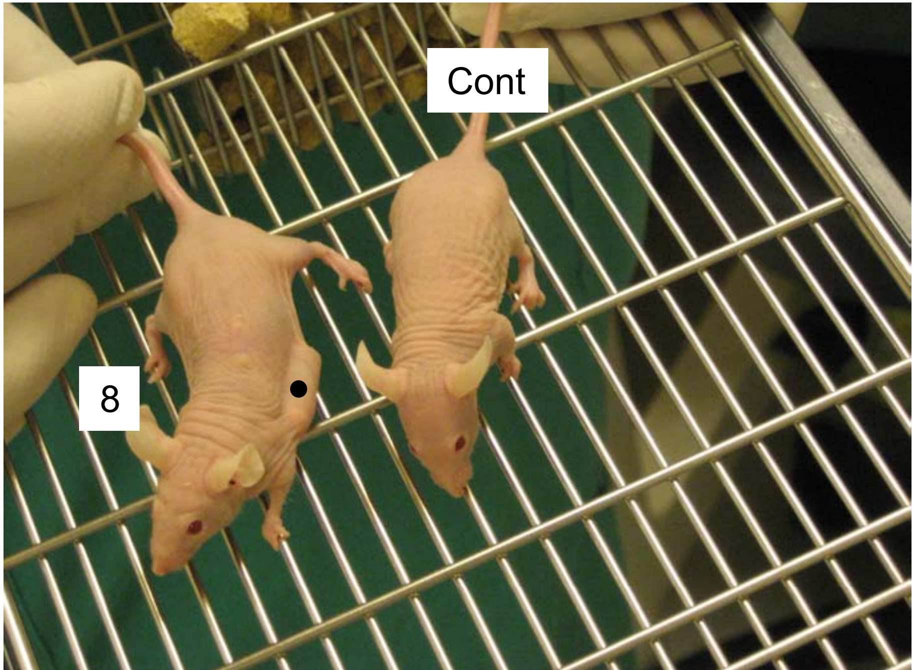
Cont
8
